# Equity-Centered Optimization of Virtual Cancer Survivorship Care

**DOI:** 10.2196/73663

**Published:** 2025-08-12

**Authors:** Abdul Moiz Ur Rab Bhatti, Muqadas Bhatti

**Affiliations:** 1 Department of Medicine, Karachi Medical and Dental College, Karachi, Pakistan; 2 Department of Public Health, Bahria University, 13 National Stadium Rd, Karachi, Pakistan, 92 3353150132

**Keywords:** cancer, follow-up, virtual, outcomes, realist evaluation, survivorship

We commend Scruton et al [1] for their realist evaluation of virtual follow-up (VFU) care experiences among breast and prostate cancer survivors. The study advances our understanding of contextual factors that influence VFU effectiveness and patient satisfaction. However, we offer critical reflections to strengthen equity-centered implementation of telehealth in survivorship care.

Although Scruton et al [1] acknowledge limitations in demographic diversity (eg, high-income, educated participants), their discussion of structural inequities remains underdeveloped. The study’s focus on individual-level mechanisms (eg, technological competence and coping styles) risks overshadowing systemic barriers that disproportionately affect marginalized groups, such as racialized communities, rural residents, and low-income populations [2]. For instance, Ontario’s reimbursement model disincentivizes low-tech telephone visits despite their significant role in bridging the digital divide [3]. Future iterations of the program theory should explicitly integrate policy-level contexts, such as equitable reimbursement structures and broadband access initiatives, to address systemic inequities.

The emphasis on provider “webside manner” and socioemotional skills is a strength that resonates with newer studies advocating for empathy training in virtual settings [4]. However, the paper’s equity discussion is surface level. It acknowledges disparities but does not engage with antiracist or decolonial approaches to telehealth, which are gaining traction in Canadian health policy. We urge future studies to intentionally recruit participants across socioeconomic, racial, and geographic spectra to test context-mechanism-outcome configurations in varied contexts.

The recommendations for optimizing VFU (eg, hybrid care models and empathy training) are pragmatic but require policy-level support. For instance, subsidized broadband access and device provision programs are important to equitable implementation [5]. Similarly, trauma-informed communication frameworks should be integrated into telehealth training curricula to address disparities in emotional support quality during virtual visits.

To conclude, the study by Scruton et al [1] advances understanding of VFU’s contextual drivers but reflects limitations common to early pandemic-era research. Future work should prioritize diverse samples, integrate implementation science frameworks, and address systemic inequities through policy-oriented recommendations.

## Abbreviations

VFU: virtual follow-up
